# Prognostic Implications of Recurrence Patterns and Time to Recurrence in Post-Hepatectomy Hepatocellular Carcinoma Recurrence: An Exploratory Analysis

**DOI:** 10.3390/curroncol33090555

**Published:** 2026-09-12

**Authors:** Jianwei Chen, Wenhao Teng, Mingji Zhang, Xiaolong Wang, Yuemin Zhu, Liting Wen, Juyi Wu, Dechun Zheng, Zhuting Fang

**Affiliations:** 1 Department of Radiology, Clinical Oncology School of Fujian Medical University, Fujian Cancer Hospital, Fuzhou 350014, China; cjjwei@163.com (J.C.); fjzhuym@163.com (Y.Z.); fjwenlt@163.com (L.W.); wjy1233q@163.com (J.W.); dechunzheng@fjmu.edu.cn (D.Z.); 2 Department of Hepatopancreatobiliary Surgery, Clinical Oncology School of Fujian Medical University, Fujian Cancer Hospital, Fuzhou 350014, China; fjtengwh@fjmu.edu.cn (W.T.); zhangmj@fjzlhospital.com (M.Z.); 3 Department of Oncology and Vascular Interventional Therapy, Clinical Oncology School of Fujian Medical University, Fujian Cancer Hospital, Fuzhou 350014, China; xlwang@fjmu.edu.cn; 4 Department of Interventional Radiology, Fujian Provincial Hospital, Fuzhou University Affiliated Provincial Hospital, Fuzhou 350001, China

**Keywords:** hepatocellular carcinoma, post-recurrence survival, recurrence pattern, BCLC stage, time to recurrence

## Abstract

Post-recurrence survival (PRS) after hepatectomy for hepatocellular carcinoma (HCC) varies widely, yet the prognostic significance of the recurrence pattern and time to recurrence (TTR) for PRS remains incompletely defined. This single-center retrospective study included 126 patients with first HCC recurrence after curative hepatectomy (2018–2024) and aimed to explore the prognostic role of recurrence pattern, classified as low-risk (oligo-recurrence: ≤3 intrahepatic nodules, each ≤3 cm) or high-risk (multiple/large intrahepatic, tumor in vein, or extrahepatic), for PRS. In the overall cohort, the recurrence pattern independently predicted PRS (hazard ratio 2.13, *p* = 0.015); the preoperative Barcelona Clinic Liver Cancer (BCLC) stage, gamma-glutamyl transferase, and total bilirubin were also independent predictors. A sensitivity analysis, including post-recurrence treatment, attenuated the hazard ratio for high-risk pattern to 1.82 (95% CI 0.93–3.55, *p* = 0.079), consistent with partial mediation. Kaplan–Meier analyses suggested an association between shorter TTR and worse overall survival (OS), but this finding is descriptive because OS measured from surgery is mathematically coupled with TTR. In an exploratory subgroup analysis of patients with early-stage primary HCC (BCLC 0/A, *n* = 98), the recurrence pattern showed a borderline association with PRS (*p* = 0.053). Late detection was associated with high-risk recurrence but did not independently affect survival. These findings highlight the prognostic value of the recurrence pattern for PRS and support the consideration of TTR as a descriptive prognostic indicator, which together may inform risk-adapted follow-up and treatment strategies.

## Simple Summary

This exploratory study of 126 patients with post-hepatectomy HCC recurrence evaluated the prognostic role of the recurrence pattern and time to recurrence (TTR). The high-risk recurrence pattern independently predicted post-recurrence survival (PRS), together with preoperative BCLC stage, GGT, and total bilirubin. An association between shorter TTR and worse overall survival (OS) was observed, but this finding is descriptive because OS measured from surgery is mathematically coupled with TTR. In an exploratory analysis of patients with early-stage primary HCC (BCLC 0/A), the recurrence pattern showed a borderline association with PRS. Late detection was associated with high-risk recurrence but did not independently affect survival.

## 1. Introduction

Hepatocellular carcinoma (HCC) is the third leading cause of cancer-related death worldwide, and surgical resection remains the mainstay of curative treatment for early-stage disease [1]. However, postoperative recurrence occurs in up to 70% of patients within 5 years, and once recurrence develops, post-recurrence survival (PRS) varies widely, ranging from a few months to several years [2]. The management of recurrent HCC is challenging because treatment options—including repeat hepatectomy, local ablation, transarterial chemoembolization (TACE), and systemic therapy—must be tailored to tumor burden, liver functional reserve, and patient performance status [3,4].

Several prior studies have identified important prognostic factors for PRS, such as recurrence pattern (intrahepatic vs. extrahepatic), time to recurrence (TTR), and liver function at the time of recurrence [5,6,7,8]. Early recurrence within 1–2 years after surgery is consistently associated with worse survival, presumably because it often reflects occult micrometastatic spread and more aggressive tumor biology [9,10]. The pattern of recurrence also carries prognostic weight: patients with oligo-recurrence (a small number of intrahepatic nodules) tend to fare better than those with multiple or large intrahepatic lesions or extrahepatic disease [11,12]. In addition, the degree of liver functional reserve at the time of recurrence, as reflected by the Child–Pugh class or albumin–bilirubin (ALBI) score, influences both the choice of feasible treatment and subsequent survival [13].

Despite these insights, several important gaps remain. First, the independent prognostic value of recurrence pattern and TTR for PRS, after accounting for established preoperative and recurrence-related factors, has not been fully characterized. Second, the association between TTR and overall survival (OS) warrants further investigation. Third, the relationship between recurrence detection mode (late vs. early) and recurrence pattern, and whether late detection independently affects survival, requires further investigation.

In this study, we aimed to explore the prognostic significance of recurrence pattern and TTR for PRS in patients with primary HCC, as well as in a subgroup of early-stage (Barcelona Clinic Liver Cancer [BCLC] stage 0/A) HCC, to investigate the association between TTR and OS, and to investigate the relationship between detection mode and recurrence pattern.

## 2. Materials and Methods

### 2.1. Study Population

This retrospective, single-center study was approved by the Institutional Review Board. Informed consent was obtained from all individual participants for the use of their clinical data.

**Figure 1:**
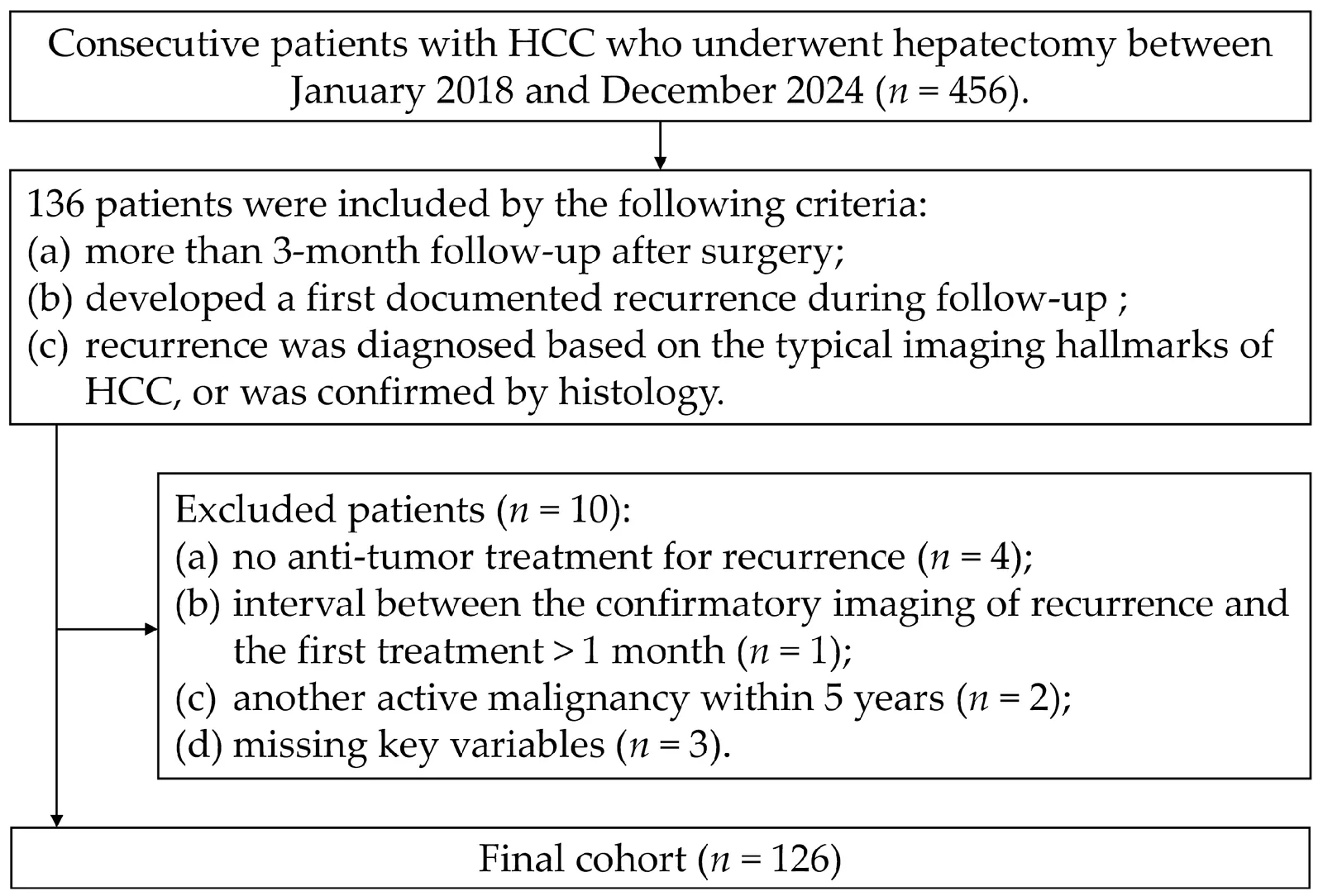
Study flow diagram.

Consecutive patients with HCC who underwent hepatectomy between January 2018 and December 2024 (*n* = 456) were screened. Among them, 136 patients developed a first documented recurrence during follow-up and met the following inclusion criteria: (a) more than 3 months of follow-up after surgery; (b) recurrence diagnosed based on typical imaging hallmarks of HCC on contrast-enhanced computed tomography (CT) or magnetic resonance imaging (MRI) [14,15,16], or confirmed by histology. Ten patients were excluded: no anti-tumor treatment for recurrence (*n* = 4); interval between confirmatory imaging and first treatment > 1 month (*n* = 1); another active malignancy within 5 years (*n* = 2); missing key variables (*n* = 3). The final cohort comprised 126 patients (Figure 1).

### 2.2. Data Collection

Data were extracted from electronic medical records and the hospital’s picture archiving and communication system. Preoperative variables included demographics (age, sex), etiology (hepatitis B virus [HBV] and hepatitis C virus [HCV]), liver function (Child–Pugh grade, presence of cirrhosis), and tumor characteristics, such as BCLC stage, serum alpha-fetoprotein (AFP) > 400 ng/mL, microvascular invasion (MVI) on pathology, and tumor differentiation grade.

Regarding recurrence-related variables, TTR was categorized as ≤1 year, 1–2 years, or >2 years from the date of surgery. Patients were initially categorized into three recurrence patterns: oligo-recurrence (≤3 intrahepatic nodules, each ≤3 cm, no tumor in vein [TIV] or extrahepatic metastasis), multiple/large intrahepatic recurrence (>3 nodules or any nodule >3 cm), and TIV/extrahepatic metastasis.

Laboratory tests collected at the time of recurrence included aspartate aminotransferase (AST), gamma-glutamyl transferase (GGT), total bilirubin, albumin, platelet count (×10^9^/L), and AFP > 400 ng/mL. The Child–Pugh grade at recurrence was also recorded. The mode of recurrence detection was classified as scheduled surveillance or late detection, based on the inter-exam interval, defined as the time from the most recent prior imaging examination to the recurrence diagnosis. Scheduled surveillance was defined as an inter-exam interval within the recommended schedule (3–6 months for the first 2 years and 6–12 months thereafter [17]). Late detection was defined as an inter-exam interval exceeding the recommended schedule. Post-recurrence treatment was categorized as curative-intent therapy (surgery or ablation) or non-curative therapy (TACE, hepatic arterial infusion chemotherapy [HAIC], radiotherapy, seed implantation, tyrosine kinase inhibitor [TKI] plus immune checkpoint inhibitor [ICI], or chemotherapy).

### 2.3. Follow-Up and Outcome

All patients were followed after recurrence with imaging and laboratory assessments until death or the censoring date (31 December 2025). The primary outcome was PRS, defined as the time from the date of recurrence diagnosis to death from any cause. The secondary outcome was OS from the date of surgery. Both PRS and OS were analyzed as time-to-event endpoints with the same censoring rules. Median follow-up was calculated using the reverse Kaplan–Meier method.

### 2.4. Statistical Analysis

Continuous variables are expressed as mean ± standard deviation (SD) or median (range), and categorical variables as counts (%). Group comparisons used the *t*-test/Mann–Whitney U test or χ^2^/Fisher’s exact test, as appropriate. The primary analysis focused on identifying independent prognostic factors for PRS. Candidate variables with univariable *p* < 0.05 were entered into the initial multivariable Cox proportional hazards model, after excluding variables with a variance inflation factor > 5 to avoid multicollinearity. Bidirectional stepwise selection based on the Akaike Information Criterion was then performed to obtain the final model. The proportional-hazards assumption was tested using Schoenfeld residuals. For the primary analysis, the three recurrence patterns were collapsed into a two-group classification (low-risk vs. high-risk). This dichotomization was finalized after inspecting the survival curves of the three original categories, and the details are presented in the Section 3.

To assess the robustness of the findings, a sensitivity analysis was performed in which post-recurrence treatment (curative-intent vs. non-curative) was added to the multivariable model for PRS. To evaluate the stability of variable selection, a bootstrap procedure was performed in which the stepwise selection process was repeated in 200 resamples, and the frequency with which each variable was retained in the final model was recorded. Standardized mean differences (SMDs) were calculated for baseline variables in the BCLC 0/A subgroup; SMD < 0.1 was considered to indicate adequate balance, 0.1–0.2 minor imbalance, and >0.2 notable imbalance. An exploratory nomogram for 12-, 24-, and 36-month PRS was built from the independent PRS predictors, with discrimination assessed by Harrell’s concordance index (C-index) and optimism corrected using 200 bootstrap resamples. Risk groups were defined by X-tile-optimized cut-offs and compared by Kaplan–Meier analysis with the log-rank test. The observed risk-group separation was derived from the same dataset used for model construction and therefore represents apparent performance, not independent validation. Given the sample size and event rate, this nomogram was considered hypothesis-generating and is presented in the Supplementary Materials. All analyses were conducted in R software (version 4.3.1), and a two-sided *p* < 0.05 was considered statistically significant.

## 3. Results

### 3.1. Baseline Characteristics of the Recurrence Cohort

**Figure 2:**
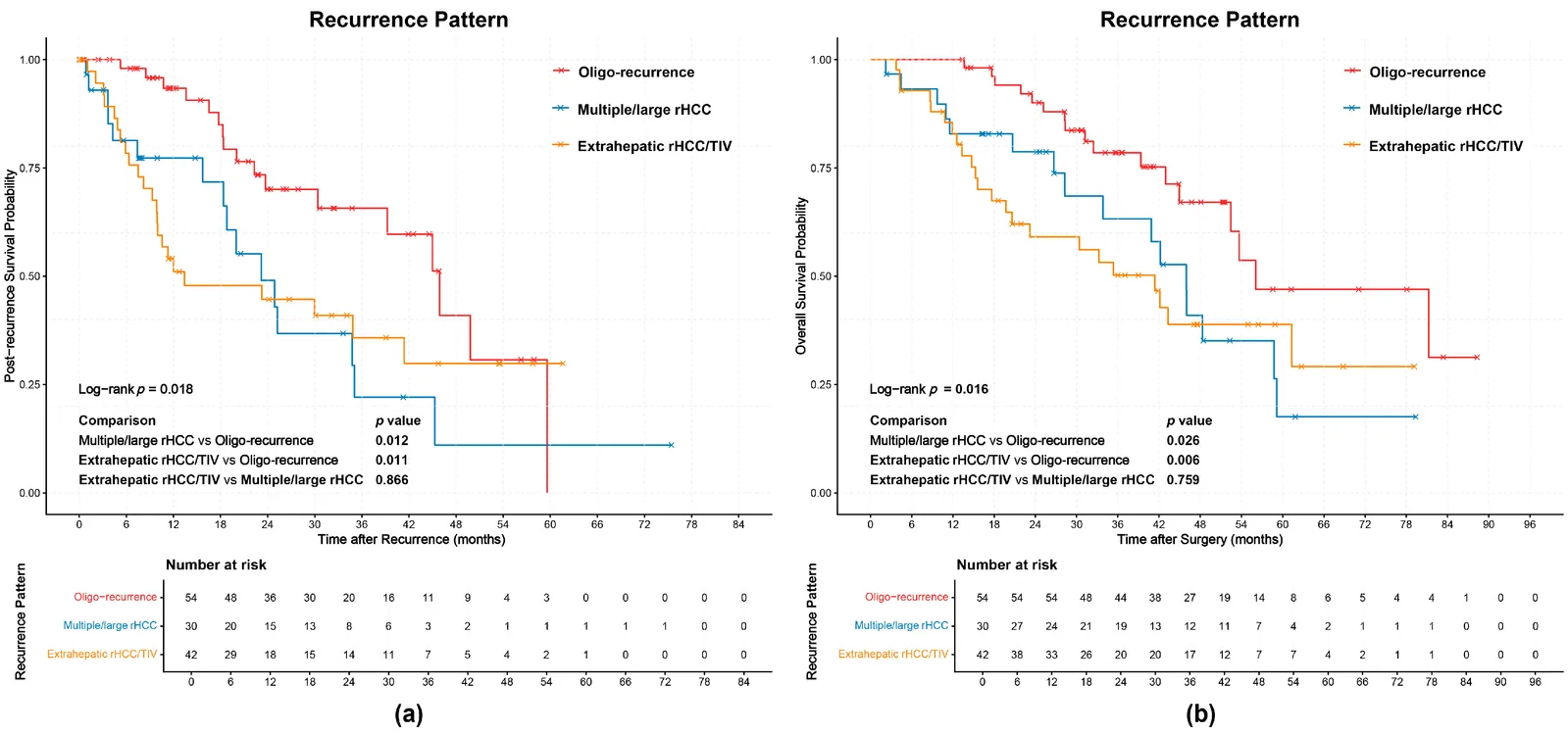
Kaplan–Meier survival curves for post-recurrence survival and overall survival stratified by the initial three-category recurrence pattern (intrahepatic oligo-recurrence, multiple/large recurrent hepatocellular carcinoma [rHCC], and extrahepatic rHCC/tumor in vein [TIV]). (**a**) Post-recurrence survival. (**b**) Overall survival.

The final cohort comprised 126 patients who developed a first HCC recurrence after hepatectomy. For descriptive purposes, the survival distribution by the three original recurrence categories is shown in Figure 2. Because the survival curves for multiple/large intrahepatic recurrence and TIV/extrahepatic recurrence were similar (log-rank *p* = 0.866 for PRS and 0.759 for OS), these two categories were combined post hoc into a single high-risk group; 54 patients (42.5%) had low-risk recurrence and 72 (57.1%) had high-risk recurrence. This dichotomization was therefore data-derived and should be interpreted as exploratory. Table 1 summarizes the baseline characteristics stratified by the final two-group classification. Compared with the low-risk pattern group, high-risk patients had a significantly shorter TTR (*p* = 0.019), higher AFP at recurrence (*p* = 0.001), higher GGT (*p* = 0.004), a higher prevalence of MVI (*p* = 0.028), and more advanced preoperative BCLC stage (*p* = 0.046).

Late detection occurred in five patients (9.3%) in the low-risk group and 19 patients (26.4%) in the high-risk group, and was significantly more frequent in the high-risk pattern group (*p* = 0.028). Overall, 24 patients (19.0%) had late detection. The mean inter-exam interval in the late detection group was 12.5 ± 10.3 months (median 7.6, range 6.0–55); only nine patients had an interval exceeding 12 months, while the remaining 15 had intervals between 6 and 12 months. In contrast, the early detection group had a mean inter-exam interval of 3.6 ± 2.0 months (median 3.1, range 1.0–11.0).

The median follow-up for PRS by the reverse Kaplan–Meier method was 27.88 months (95% CI: 23.97–34.75), and for OS, 45.1 months (95% CI: 39.77–51.6).

### 3.2. Overall and Subgroup Survival Analysis

**Figure 3:**
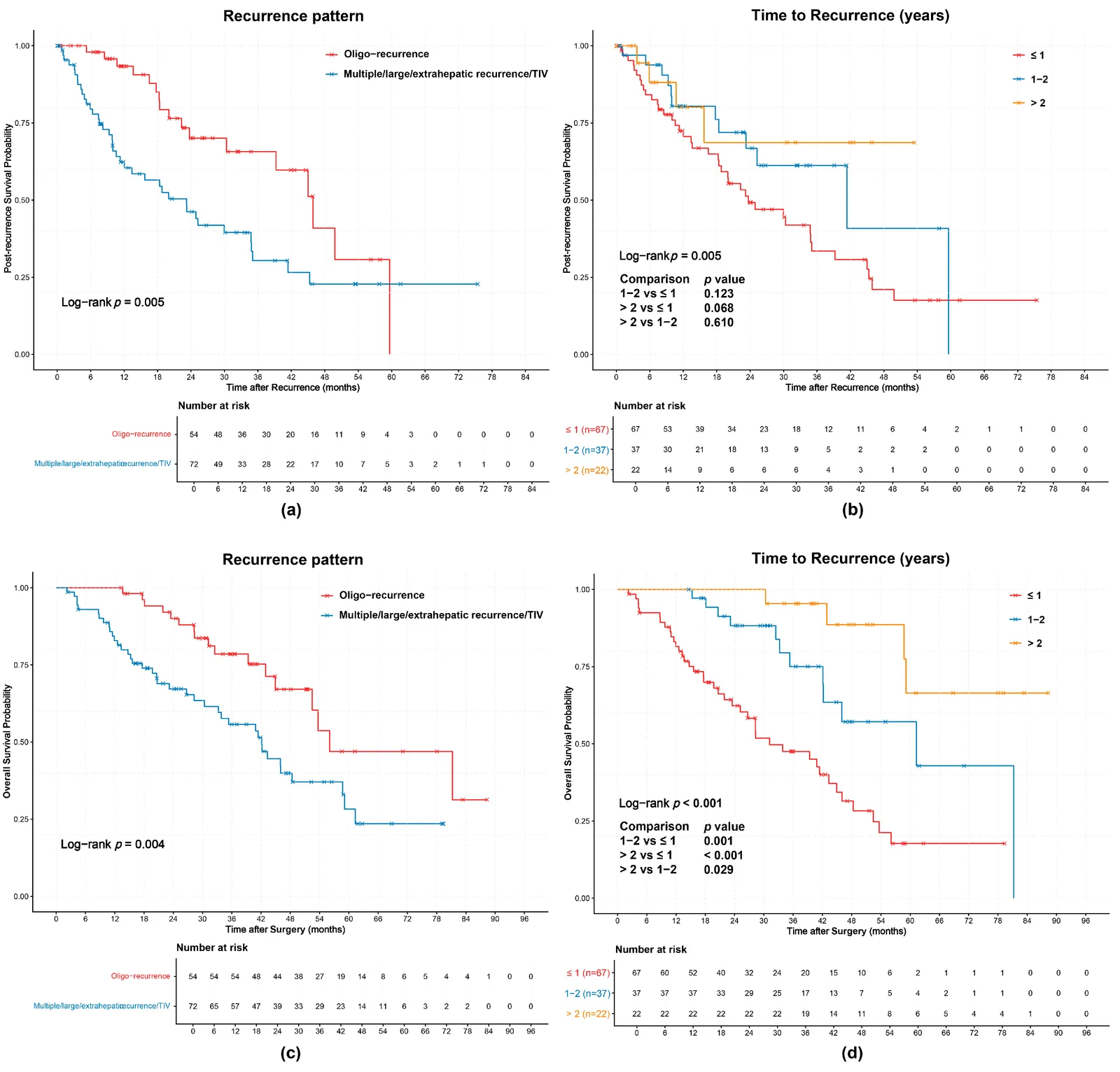
Kaplan–Meier survival curves for post-recurrence survival and overall survival stratified by recurrence pattern and time to recurrence (TTR). (**a**) Post-recurrence survival (PRS) according to recurrence pattern (low-risk vs. high-risk). (**b**) PRS according to TTR (≤1 year, 1–2 years, >2 years). (**c**) Overall survival (OS) according to recurrence pattern. (**d**) OS according to TTR. Corresponding log-rank *p* values are shown in each panel. TIV, tumor in vein.

At the end of follow-up, 56 all-cause deaths were recorded. The median PRS for the entire cohort was 34.78 months (95% confidence interval [CI]: 23.19–45.29). Patients in the low-risk recurrence pattern group had a significantly longer median PRS than those in the high-risk group (45.88 months, 95% CI 39.25–not reached [NR] vs. 23.19 months, 95% CI 12.01–35.04, log-rank *p* = 0.005; Figure 3a). When stratified by TTR, median PRS decreased stepwise: 23.70 months for recurrence ≤ 1 year, 41.39 months for 1–2 years, and NR for >2 years (log-rank *p* = 0.084; Figure 3b).

The median OS for the entire cohort was 48.33 months (95% CI 42.20–61.30). By recurrence pattern, the low-risk group had a median OS of 56.07 months (95% CI 52.46–NR), compared with 42.10 months (95% CI 33.23–59.13) in the high-risk group (log-rank *p* = 0.004; Figure 3c). Similarly, OS showed a clear gradient across TTR categories: 31.20 months (95% CI 25.17–46.00) for ≤ 1 year, 61.30 months (95% CI 42.20–NR) for 1–2 years, and not reached for >2 years (log-rank *p* < 0.001; Figure 3d).

### 3.3. Independent Prognostic Factors for PRS

The results of univariable and multivariable Cox regression for PRS are summarized in Table 2. The proportional-hazards assumption was not violated (global Schoenfeld test *p* = 0.176). In the multivariable analysis, four factors were identified as independent adverse prognostic factors: high-risk recurrence pattern (hazard ratio [HR] = 2.13, 95% CI 1.16–3.92, *p* = 0.015), higher GGT (HR = 1.00 per U/L, *p* = 0.027), higher total bilirubin (HR = 1.05 per μmol/L, *p* = 0.002), and more advanced preoperative BCLC stage (HR = 1.71, 95% CI 1.12–2.63, *p* = 0.014). Although significant in univariable analysis, TTR and AST did not retain significance in the multivariable model. Late detection also did not independently predict PRS (univariable *p* = 0.282). The multivariable model for PRS achieved a C-index of 0.767 (95% CI 0.703–0.831), with a bootstrap-corrected C-index of 0.757.

A sensitivity analysis, including post-recurrence treatment (curative-intent vs. non-curative) as an additional covariate, was performed. In this model, the HR for high-risk recurrence pattern was attenuated from 2.13 (95% CI 1.16–3.92, *p* = 0.015) to 1.82 (95% CI 0.93–3.55, *p* = 0.079), consistent with treatment acting as a partial mediator. The effect remained in the same direction, suggesting that the recurrence pattern may retain prognostic information beyond treatment allocation.

Bootstrap stability assessment (200 resamples) indicated that the four independent predictors were retained in the majority of resamples: total bilirubin, 86%; GGT, 71%; recurrence pattern, 67%; and preoperative BCLC stage, 64%. These results support the robustness of the final model.

### 3.4. Subgroup Analysis of Patients with Early-Stage Primary HCC (BCLC 0/A)

To assess the prognostic impact of recurrence pattern in a setting with homogeneous initial tumor burden, we performed a pre-specified exploratory subgroup analysis of patients with BCLC stage 0 or A primary HCC (*n* = 98). Of these, 41 had oligo-recurrence, and 57 had high-risk recurrence. Table 3 presents the baseline characteristics of this subgroup. Preoperative and pathological variables—including age, sex, etiology, Child–Pugh grade, AFP, cirrhosis, MVI, and tumor differentiation—did not differ significantly between the two recurrence pattern groups. Most of the variables had SMD < 0.2, indicating reasonable balance or minor imbalance between the two recurrence pattern groups. However, AFP > 400 ng/mL (SMD = −0.442) and MVI (SMD = −0.201) showed notable imbalance. In contrast, at the time of recurrence, the high-risk group showed significantly higher rates of AFP > 400 ng/mL (*p* = 0.008), higher GGT (*p* = 0.021), more frequent late detection (*p* = 0.021), and a lower proportion of patients receiving curative-intent surgery/ablation (*p* < 0.001).

**Figure 4:**
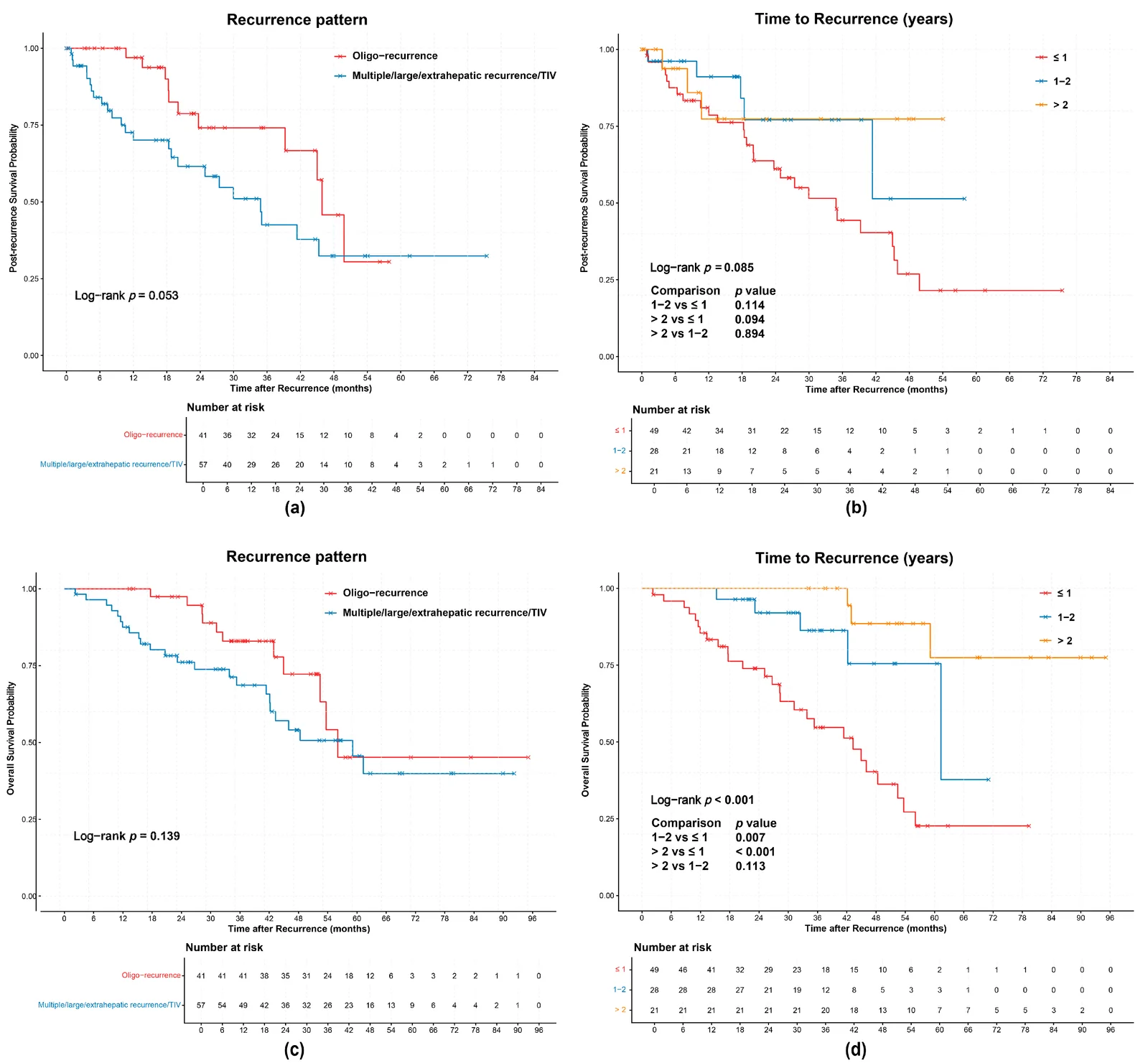
Kaplan–Meier survival curves for the BCLC 0/A subgroup (*n* = 98). (**a**) Post-recurrence survival (PRS) according to recurrence pattern (low-risk vs. high-risk). (**b**) PRS according to time to recurrence (TTR: ≤1 year, 1–2 years, >2 years). (**c**) Overall survival (OS) according to recurrence pattern. (**d**) OS according to TTR. Log-rank *p* values are shown in each panel. Pairwise comparisons for TTR categories are provided in the text. BCLC, Barcelona Clinic Liver Cancer; PRS, post-recurrence survival; OS, overall survival; TTR, time to recurrence.

Kaplan–Meier analysis of this subgroup showed that PRS was not significantly different between low-risk and high-risk patterns, although a borderline trend was observed (median PRS: 45.9 months vs. 35.8 months, log-rank *p* = 0.053) (Figure 4a). When stratified by TTR, PRS exhibited a non-significant gradient (overall log-rank *p* = 0.085) (Figure 4b). OS did not differ significantly by recurrence pattern (log-rank *p* = 0.139) (Figure 4c). In contrast, OS showed a highly significant gradient across TTR categories (overall log-rank *p* < 0.001; pairwise: 1–2 years vs. ≤1 year, *p* = 0.007; >2 years vs. ≤1 year, *p* < 0.001; >2 years vs. 1–2 years, *p* = 0.113) (Figure 4d).

## 4. Discussion

In this exploratory study of 126 patients with post-hepatectomy HCC recurrence, we demonstrated that recurrence pattern, preoperative BCLC stage, GGT, and total bilirubin were independent predictors of PRS. Kaplan–Meier analyses suggested an association between shorter TTR and worse OS. We further constructed an exploratory nomogram for PRS risk stratification, presented in the Supplementary Material. In the BCLC 0/A subgroup, the prognostic effect of recurrence pattern on PRS was less pronounced (log-rank *p* = 0.053). Late detection was more frequent in the high-risk group but did not independently affect survival. These findings highlight the prognostic value of the recurrence pattern for PRS and the descriptive association of TTR with OS, suggesting that both factors merit consideration in post-recurrence risk assessment, albeit in different analytical frameworks.

### 4.1. Recurrence Timing and Pattern

Several large series have demonstrated that early recurrence (usually within 1–2 years) is associated with worse PRS [2,6,7]. Consistent with this, we observed a stepwise improvement in median PRS with longer TTR (23.7, 41.4, and NR). However, TTR did not retain independent significance for PRS in the multivariable model (*p* = 0.093). This discrepancy likely reflects collinearity between TTR, recurrence pattern, and BCLC stage: early recurrences are predominantly of the high-risk pattern and arise from more advanced primary tumors [9,10], and once these factors are accounted for, TTR adds limited independent information for PRS.

This interplay between recurrence timing and biology is further illustrated by our findings on MVI. Although MVI was more frequent in the high-risk pattern group and was a significant predictor in the univariable analysis, it was not retained in multivariable models. This suggests that the prognostic information carried by MVI is largely captured by the high-risk recurrence pattern and advanced BCLC stage—both of which remained independently associated with prognosis [18]. The relatively small number of MVI-negative patients in our cohort may also have limited the statistical power to detect an independent effect. Thus, while MVI is a well-established risk factor for recurrence after primary hepatectomy [19,20], its direct independent effect on PRS requires validation in larger cohorts.

Regarding the recurrence pattern itself, we found that intrahepatic oligo-recurrence carries a better prognosis than multiple/large intrahepatic nodules or extrahepatic spread [12]. Although the dichotomization was finalized after inspecting the survival curves rather than being defined a priori, it is consistent with the clinical concept that recurrences amenable to curative local therapy portend a better prognosis than those that are not [4].

PRS and OS both showed a gradient across TTR categories, although only OS reached statistical significance. This may partly reflect the mathematical coupling between TTR and OS (OS = TTR + PRS), since OS is measured from surgery and TTR is the elapsed time from surgery to recurrence. Nevertheless, the observation that early recurrence (TTR ≤ 1 or ≤ 2 years) is an important adverse prognostic factor is consistent with most previous studies and aligns with our OS findings [21,22].

### 4.2. Subgroup Analysis of BCLC 0/A Patients

In the BCLC 0/A subgroup, preoperative characteristics were assessed using SMDs, which suggested reasonable balance or minor imbalance for most variables, with notable imbalance for AFP and MVI. The observed PRS difference by recurrence pattern was of borderline significance (*p* = 0.053), but this subgroup analysis is exploratory and should not be interpreted as evidence of effect modification. Because no formal interaction test was performed, we cannot determine whether the prognostic effect of recurrence pattern truly differs between early-stage and more advanced primary disease, or whether the reduced significance reflects limited statistical power. In contrast, TTR remained a clear gradient for OS, demonstrating that even in early-stage primary disease, the timing of recurrence may reflect long-term outcomes [23,24]. These findings should be interpreted with caution, given the sample size.

### 4.3. Liver Function Markers

Liver function at recurrence has received less attention as a direct prognostic factor compared with tumor-related variables. The ALBI score and Child–Pugh grade remain cornerstones for assessing hepatic reserve and guiding therapy in recurrent HCC [13,25]. Beyond these composite scores, individual liver enzymes also carry prognostic information. In our analysis, GGT independently predicted shorter PRS, consistent with its known association with aggressive tumor biology [26,27]. Total bilirubin also independently predicted shorter PRS. Although bilirubin is a component of both Child–Pugh and ALBI, its independent prognostic value suggests that it captures more than chronic excretory dysfunction—it may also reflect the systemic burden of aggressive recurrence, particularly when elevated without end-stage liver disease [28].

### 4.4. Impact of Detection Mode and Surveillance Interval

The mode of detection (late vs. early) did not independently predict survival in the present cohort. This finding may appear to contrast with studies advocating intensive surveillance [29]. However, several considerations are relevant. Most patients (81.0%) were in the early detection group, with a median inter-exam interval of 3.1 months, and even among the 24 late-detected cases, the majority (15/24) had intervals of only 6–12 months—a modest delay unlikely to substantially alter tumor stage or treatment options. More importantly, late detection was significantly associated with the high-risk recurrence pattern. However, our observational data cannot distinguish whether this association reflects more aggressive tumor biology, reduced surveillance adherence, barriers to care, or simply that longer imaging intervals allow recurrences to progress before detection. These alternative explanations are not mutually exclusive.

These observations reinforce the notion that once recurrence occurs, the intrinsic biology of the recurrence—as captured by pattern, TTR, and liver function—is more important than the timing of its discovery [30]. This does not diminish the value of regular surveillance, which remains essential for identifying treatable oligo-recurrences. Rather, it emphasizes that post-recurrence treatment decisions should be guided by the characteristics of the recurrence itself, not by whether it was detected early or late. We therefore support current guideline recommendations for regular surveillance [14,17].

### 4.5. Methodological Considerations Regarding Post-Recurrence Treatment

An important methodological consideration concerns the handling of post-recurrence treatment. In our cohort, treatment allocation was strongly determined by recurrence pattern: curative-intent therapy was administered to 63.0% of patients with low-risk recurrence but only 13.9% of those with high-risk recurrence. From a causal inference perspective, post-recurrence treatment therefore lies on the causal pathway between recurrence pattern and survival, acting as a mediator rather than a confounder. Adjusting for a mediator introduces overadjustment bias, which can attenuate or obscure the total effect of the exposure on the outcome. For this reason, we chose not to include post-recurrence treatment in our primary models, in accordance with established epidemiological principles. We acknowledge that this approach does not allow us to fully disentangle the direct effect of the recurrence pattern from that mediated through treatment allocation, and future studies employing formal mediation analysis are needed to address this question.

As a sensitivity analysis, we additionally adjusted for post-recurrence treatment in the multivariable model. The HR for the high-risk recurrence pattern was attenuated from 2.13 to 1.82 (*p* = 0.079), consistent with partial mediation by treatment allocation. Although the association became borderline, the direction of the effect was preserved, supporting the interpretation that the recurrence pattern carries prognostic information beyond treatment assignment.

## 5. Limitations

Several limitations should be acknowledged. First, this single-center retrospective study had a moderate sample size (*n* = 126), and the BCLC 0/A subgroup analysis (*n* = 98) was exploratory with limited statistical power. With 11 candidate variables entered into the multivariable model and 56 events, the events-per-variable ratio was approximately 5.1, below the conventional threshold of 10. However, bootstrap stability assessment (200 resamples) showed that all four independent predictors were retained in more than 60% of resamples, supporting the robustness of the final model despite the limited sample size. Second, post-recurrence treatment was not included in the primary models because it acts as a mediator rather than a confounder; a sensitivity analysis, including treatment attenuation, did not eliminate the association between recurrence pattern and PRS. Third, the nomogram was exploratory and lacked external validation, and the X-tile-derived risk stratification represents apparent performance only. Fourth, our cohort was predominantly HBV-related HCC from a single center, limiting generalizability to populations with other etiologies. Fifth, excluding patients without active treatment or with treatment delay may have introduced selection bias and limits the applicability. Sixth, the relatively small number of MVI-negative patients limited statistical power to assess the independent effect of MVI. Seventh, late detection was defined solely by the inter-exam interval without incorporating symptomatic presentation, which may have caused misclassification; future prospective studies should include standardized symptom assessment.

## 6. Conclusions

This single-center exploratory study identified recurrence pattern as an independent predictor of PRS after HCC recurrence, with preoperative BCLC stage, GGT, and total bilirubin as additional independent predictors. In line with the study’s focus on recurrence patterns and TTR, the Kaplan–Meier analyses suggested an association between shorter TTR and worse OS, although this finding is reported descriptively because OS measured from surgery is mathematically coupled with TTR. In an exploratory subgroup analysis of patients with early-stage primary HCC (BCLC 0/A), the recurrence pattern showed a borderline association with PRS. Late detection was associated with high-risk recurrence but did not independently affect survival. These findings highlight the prognostic value of recurrence patterns for PRS and support the consideration of TTR as a descriptive prognostic indicator, which together may inform risk-adapted follow-up and treatment strategies.

## Figures and Tables

**Table 1:** Baseline characteristics of the study cohort stratified by recurrence pattern.

Variable	Oligo-Recurrence (*n* = 54)	Multiple/Large/Extrahepatic Recurrence/TIV (*n* = 72)	*p* Value
Data at the time of recurrence			
Male	50 (92.6)	69 (95.8)	0.461
Age (years)	54.54 ± 11.35	55.61 ± 10.74	0.589
Time to recurrence (years)			0.019
≤1	21 (38.9)	46 (63.9)	
1–2	20 (37.0)	17 (23.6)	
˃2	13 (24.1)	9 (12.5)	
Late detection	5 (9.3)	19 (26.4)	0.028
Post-recurrence treatment			<0.001
surgery/ablation	34 (63.0)	10 (13.9)	
other treatments	20 (37.0)	62 (86.1)	
AFP ˃ 400 ng/mL	4 (7.4)	24 (33.3)	0.001
Child–Pugh			0.136
A	53 (98.1)	65 (90.3)	
B	1 (1.9)	7 (9.7)	
ALT (U/L)	41.96 ± 71.21	40.99 ± 30.97	0.285
AST (U/L)	38.57 ± 26.39	50.26 ± 40.84	0.276
GGT (U/L)	68.85 ± 69.74	112.41 ± 117.98	0.004
Total bilirubin (μmol/L)	15.29 ± 6.20	18.02 ± 13.16	0.126
Albumin (g/L)	40.40 ± 4.22	39.17 ± 4.94	0.145
PLT (×10^9^)	155.80 ± 50.99	162.84 ± 68.36	0.886
Preoperative and pathological data			
HBV infection	44 (81.5)	60 (83.3)	0.973
BCLC stage			0.046
0	4 (7.4)	2 (2.8)	
A	37 (68.5)	50 (69.4)	
B	13 (24.1)	13 (18.1)	
C	0 (0.0)	7 (9.7)	
Child–Pugh			˃0.999
A	54 (100.0)	71 (98.6)	
B	0 (0.0)	1 (1.4)	
AFP ˃ 400 ng/mL	14 (25.9)	30 (41.7)	0.100
Tumor differentiation degree			0.798
Poorly	10 (18.5)	17 (23.6)	
Moderately	40 (74.1)	49 (68.1)	
Well	4 (7.4)	6 (8.3)	
MVI	34 (63.0)	59 (81.9)	0.028
Cirrhosis	31 (57.4)	29 (40.3)	0.085

Data are presented as n (%) or mean ± standard deviation. Abbreviations: HCC, hepatocellular carcinoma; TIV, tumor in vein (including portal vein or hepatic vein tumor thrombus); AFP, alpha-fetoprotein; ALT, alanine aminotransferase; AST, aspartate aminotransferase; GGT, gamma-glutamyl transferase; PLT, platelet count; MVI, microvascular invasion; HBV, hepatitis B virus; BCLC, Barcelona Clinic Liver Cancer. Late detection: recurrence identified by unscheduled surveillance, with an inter-exam interval > 6 months in the first 2 years or > 12 months thereafter.

**Table 2:** Univariable and multivariable Cox regression analyses for post-recurrence survival.

Variable	Univariable Analysis		Multivariable Analysis	
	HR (95% CI)	*p* Value	HR (95% CI)	*p* Value
Data at the time of recurrence				
Male	1.50 (0.47–4.84)	0.496		
Age (years)	0.99 (0.96–1.01)	0.315		
Time to recurrence (≤1/1–2/˃2 years)	0.61 (0.40–0.94)	0.026	0.66 (0.41–1.07)	0.093
Late detection	1.44 (0.74–2.80)	0.282		
Recurrence pattern (Multiple/large/extrahepatic recurrence/TIV vs. oligo-recurrence)	2.45 (1.38–4.33)	0.002	2.13 (1.16–3.92)	0.015
Post-recurrence treatment (surgery/ablation vs. other treatments)	0.39 (0.20–0.76)	0.005		
Child–Pugh	1.20 (0.37–3.87)	0.757		
AFP ˃ 400 ng/mL	1.50 (0.82–2.74)	0.189		
AST (U/L)	1.01 (1.01–1.02)	˂0.001	1.01 (1.00–1.02)	0.055
GGT (U/L)	1.01 (1.00–1.01)	˂0.001	1.00 (1.00–1.01)	0.027
Total bilirubin (μmol/L)	1.05 (1.03–1.08)	˂0.001	1.05 (1.02–1.08)	0.002
ALT (U/L)	1.00 (1.00–1.01)	0.044		
Albumin (g/L)	0.97 (0.91–1.04)	0.444		
PLT (×10^9^)	1.00 (1.00–1.01)	0.441		
Preoperative and pathological data				
AFP ˃ 400 ng/mL	1.20 (0.70–2.07)	0.515		
BCLC stage	2.16 (1.43–3.26)	˂0.001	1.71 (1.12–2.63)	0.014
Child–Pugh	7.97 (1.04–60.91)	0.046		
HBV infection	1.55 (0.66–3.62)	0.315		
Tumor differentiation degree (poorly/moderately/well)	0.51 (0.30–0.87)	0.013	0.64 (0.35–1.16)	0.139
MVI	2.50 (1.26–4.97)	0.009		
Cirrhosis	1.30 (0.77–2.22)	0.326		

Abbreviations: HR, hazard ratio; CI, confidence interval; AST, aspartate aminotransferase; GGT, gamma-glutamyl transferase; ALT, alanine aminotransferase; AFP, alpha-fetoprotein; MVI, microvascular invasion; HBV, hepatitis B virus; BCLC, Barcelona Clinic Liver Cancer; TIV, tumor in vein; PLT, platelet count. Variables with univariable *p* < 0.05 were entered into the initial multivariable Cox model; the final model was selected by bidirectional stepwise selection based on the Akaike Information Criterion.

**Table 3:** Baseline characteristics of the preoperative BCLC stage 0/A subgroup.

Variable	Oligo-Recurrence (*n* = 41)	Multiple/Large Recurrence (*n* = 57)	SMD	*p* Value
Data at the time of recurrence				
Male	39 (95.1%)	52 (91.2%)	0.155	0.696
Age (years)	54.58 ± 11.43	55.26 ± 10.45	−0.062	0.772
Time to recurrence (years)			0.194	0.590
≤1	18 (43.9%)	31 (54.4%)		
1–2	13 (31.7%)	15 (26.3%)		
˃2	10 (24.4%)	11 (19.3%)		
Late detection	4 (9.8%)	18 (31.6%)	−0.560	0.021
Post-recurrence treatment			0.986	<0.001
surgery/ablation	24 (58.5%)	9 (15.8%)		
other treatments	17 (41.5%)	48 (84.2%)		
AFP ˃ 400 ng/mL	4 (9.8%)	20 (35.1%)	−0.637	0.008
Child–Pugh			−0.384	0.134
A	40 (97.6%)	50 (87.7%)		
B	1 (2.4%)	7 (12.3%)		
ALT (U/L)	43.46 ± 80.23	45.82 ± 51.31	−0.035	0.235
AST (U/L)	37.98 ± 30.57	57.39 ± 68.46	−0.366	0.303
GGT (U/L)	59.98 ± 59.76	108.89 ± 115.99	−0.530	0.021
Total bilirubin (μmol/L)	15.11 ± 5.88	23.65 ± 31.70	−0.375	0.129
Albumin (g/L)	40.75 ± 4.45	39.17 ± 5.58	0.314	0.136
PLT (×10^9^)	158.17 ± 46.75	156.29 ± 69.86	0.032	0.506
Preoperative and pathological data				
HBV infection	33 (80.5%)	49 (86.0%)	−0.147	0.655
BCLC stage			−0.253	0.233
0	4 (9.8%)	2 (3.5%)		
A	37 (90.2%)	55 (96.5%)		
Child–Pugh			0.000	˃0.999
A	41 (100.0%)	57 (100.0%)		
B	0 (0.0)	0 (0.0)		
AFP ˃ 400 ng/mL	9 (22.0%)	24 (42.1%)	−0.442	0.062
Tumor differentiation degree			0.021	0.424
Poorly	8 (19.5%)	15 (26.3%)		
Moderately	31 (75.6%)	36 (63.2%)		
Well	2 (4.9%)	6 (10.5%)		
MVI	28 (68.3%)	44 (77.2%)	−0.201	0.452
Cirrhosis	21 (51.2%)	29 (50.9%)	0.007	˃0.999

Data are presented as n (%) or mean ± standard deviation. Abbreviations: HCC, hepatocellular carcinoma; AFP, alpha-fetoprotein; ALT, alanine aminotransferase; AST, aspartate aminotransferase; GGT, gamma-glutamyl transferase; PLT, platelet count; MVI, microvascular invasion; HBV, hepatitis B virus; BCLC, Barcelona Clinic Liver Cancer; SMD, standardized mean difference. Late detection: recurrence identified by unscheduled surveillance, with an inter-exam interval > 6 months in the first 2 years or > 12 months thereafter.

## Data Availability

The data presented in this study are available on request from the corresponding author.

## Supplementary Materials

The following supporting information can be downloaded at: https://www.mdpi.com/article/10.3390/curroncol33090555/s1, Figure S1: (a) Nomogram for predicting 12-, 24-, and 36-month post-recurrence survival after hepatocellular carcinoma (HCC) recurrence. *Assessed at recurrence. #Assessed preoperatively. (b) Risk stratification based on the nomogram. Kaplan–Meier post-recurrence survival curves for the three risk groups derived from X-tile-optimized cut-off values of total nomogram points. BCLC, Barcelona Clinic Liver Cancer; GGT, gamma-glutamyl transferase; PRS, post-recurrence survival.

## Abbreviations

AFP, alpha-fetoprotein; ALBI, albumin–bilirubin; AST, aspartate aminotransferase; BCLC, Barcelona Clinic Liver Cancer; CI, confidence interval; CT, computed tomography; C-index, concordance index; GGT, gamma-glutamyl transferase; HAIC, hepatic arterial infusion chemotherapy; HBV, hepatitis B virus; HCC, hepatocellular carcinoma; HCV, hepatitis C virus; HR, hazard ratio; ICI, immune checkpoint inhibitor; MRI, magnetic resonance imaging; MVI, microvascular invasion; NR, not reached; OS, overall survival; PRS, post-recurrence survival; SD, standard deviation; TACE, transarterial chemoembolization; TIV, tumor in vein; TKI, tyrosine kinase inhibitor; TTR, time to recurrence.
