# Supplementary material for: Prognostic Implications of Recurrence Patterns and Time to Recurrence in Post-Hepatectomy Hepatocellular Carcinoma Recurrence: An Exploratory Analysis
Source: Curr Oncol. 2026 Sep 12;33(9):555. doi: 10.3390/curroncol33090555 (PMC13605822; doi:10.3390/curroncol33090555)
Supplement: Supplementary file 1 [file curroncol-33-00555-s001.zip › curroncol-4497764-supplementary.pdf]

Exploratory nomogram for PRS prediction

For exploratory purposes, a nomogram based on the four independent PRS predictors (recurrence pattern, GGT, total bilirubin, and preoperative BCLC stage) was constructed (**Figure S1a**). Optimal cut-off values for total nomogram points (24.46 and 40.93) were determined using X-tile software, which stratified patients into three risk tiers: low-risk (≤ 24 points, n = 55), intermediate-risk (25–40 points, n = 53), and high-risk (> 40 points, n = 16). Median PRS was 45.9 months (95% CI 45.0–not reached) in the low-risk group, 24.9 months (15.7–35.0) in the intermediate-risk group, and 5.3 months (3.2–not reached) in the high-risk group (log-rank $p < 0.001$; **Figure S1b**). Given the limited sample size and the number of events, this nomogram should be interpreted as exploratory and requires external validation.

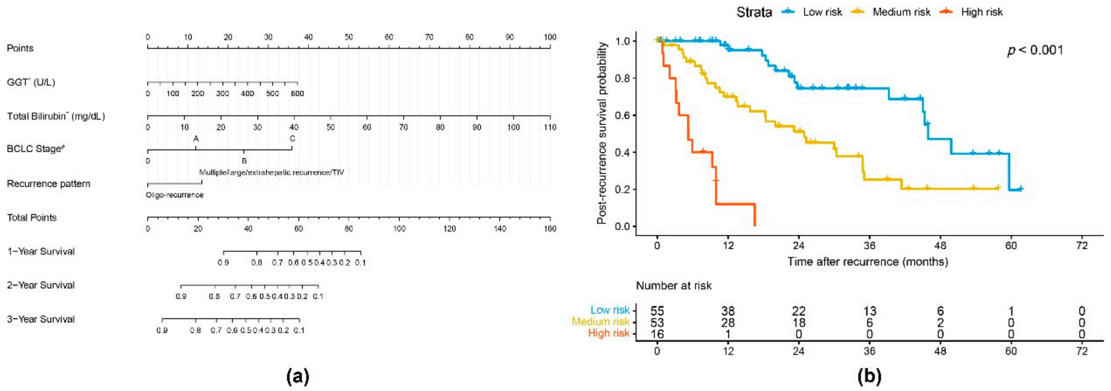

Figure S1. (**a**) Nomogram for predicting 12-, 24-, and 36-month post-recurrence survival after hepatocellular carcinoma (HCC) recurrence. *Assessed at recurrence. #Assessed preoperatively. (**b**) Risk stratification based on the nomogram. Kaplan–Meier post-recurrence survival curves for the three risk groups derived from X-tile-optimized cut-off values of total nomogram points. BCLC, Barcelona Clinic Liver Cancer; GGT, gamma-glutamyl transferase; PRS, post-recurrence survival.
